# Profile of 50 m Sprinting: The Influence of Carbon-Plated Spikes on Maximum-Velocity Performance

**DOI:** 10.3390/s25071979

**Published:** 2025-03-22

**Authors:** Krzysztof Mackala, Michal Krzysztofik, Adrian Weber, Dariusz Mroczek, Adam Zajac

**Affiliations:** 1 Department of Track and Field, Wroclaw University of Health and Sport Sciences, al. Ignacego Jana Paderewskiego 35, 51-612 Wrocław, Poland; adrian.webr@awf.wroc.pl; 2 Institute of Sport Sciences, The Jerzy Kukuczka Academy of Physical Education in Katowice, Mikołowska 72A, 40-065 Katowice, Poland; m.krzysztofik@awf.katowice.pl (M.K.); a.zajac@awf.katowice.pl (A.Z.); 3 Department of Biological and Motor Bases of Sports, Wroclaw University of Health and Sport Sciences, al. Ignacego Jana Paderewskiego 35, 51-612 Wrocław, Poland; dariusz.mroczek@awf.wroc.pl

**Keywords:** sprinting, kinematics, carbon-plated spikes, maximum speed, elastic energy

## Abstract

**Highlights:**

- The 2021 Tokyo Olympics Games ended with fantastic sprints and hurdles results (world record in the 400 m hurdles). Many were concerned about whether the results were influenced by athletes’ use of new carbon-plated spikes.
- This study compares the effectiveness of classic carbon-plated fiber spikes in improving maximum sprinting speed. The analysis concerns the variability of sprinting kinematic parameters.
- The four 50 m full-out sprints did not show significant differences between the kinematic parameters considering the performance of classic Nike and carbon-plated Nike ZoomX Flymax spikes. This means that in maximum speed training, the effectiveness of both spikes is the same.

**Abstract:**

The main goal of this study was to determine whether the type of spike can influence the final sprint result by comparing step by step the kinematics of four 50-m sprints. Twelve well-trained junior sprinters (ages 17–19) from the Polish National Team (ranging from 100 to 400 m) participated in the study, with personal bests in the 100-m sprint of 10.70 ± 0.19 s. The OptoJump Next-Microgate sensor measurement system (Optojump, Bolzano, Italy) was used to measure the essential kinematic sprinting variables. Following the sprint distance, photocells were placed on the track at the start, at 10 m, at 20 m, at 30 m, and at the finish (50 m). Fifty-meter sprints were completed alternately, two with classic and two with the carbon-plated spikes. For every sprinter, the order in which the spikes were chosen was randomized. To better understand the problem of variability in kinematic parameters, in addition to the actual statistics, the profile analysis process was applied. The analysis of the four 50 m sprints did not show significant differences between the kinematic parameters considering runs in both the classic Nike and carbon-plated Nike ZoomX Flymax spikes. It may be suggested that spikes’ sole bending stiffness may not affect short-distance (up to 50–60 m) sprinting performance. From a practical point of view, training focused on maximum speed development can be carried out with both classic and carbon-plated spikes. Finally, our experiment can guide the preparation of a research methodology that assesses the effect of carbon-plated spikes on prolonged sprinting, e.g., 200–400 m.

## 1. Introduction

One of the most critical skills in short sprints (60–200 m) is to develop the highest possible maximum speed [1,2,3,4,5]. In the case of longer sprints up to 400 m [6,7,8], the essence is to maintain developed speed with increasing fatigue and its mechanical and physiological effects. According to Yousif et al. [9], Zauhal et al. [6], Young and Choice [10], Plevnik et al. [11], Vazel [7], and Haugen and Buchheit [12], these variables are related to the degree of performance, gender, morphological and physiological characteristics, and the level of motor skills. The sum of all these elements in a comprehensive training process can determine the result in sprinting [13]. However, giving sprinters access to contemporary training methods, approved physiological ergogenic aids, and post-exercise recovery plans [14] is no longer sufficient to enhance their sprint performance. New technologies are the second area influencing these results [15,16]. This applies to training equipment, state-of-the-art diagnostic tools in sports, and most importantly, personal equipment, such as running shoes and spikes.

In recent years (2021–2024), sprinting outcomes have significantly improved since the utilization of super spikes [17,18] with a carbon fiber sole and PEBAX [17,18,19]. After the 2021 Olympics in Tokyo and the results obtained in sprint and hurdle events (a world record in the 400 m hurdles) [17,18], much was written about whether the results were influenced by new training or using new spikes with carbon soles. Since 2021, four sports shoe companies, Nike, Puma, Adidas, and New Balance, introduced a new type of running spike, the carbon-plated spike, to the market. The case directly concerned four sports shoe companies, which introduced a new type of running spike, the carbon-plated spike, on the market. However, the most frequently used spikes were the Nike Zoom Max Fly. A detailed description of which spike brands entered the sports market can be found in the article by Healey et al. [17].

Only two articles [17,18] have dealt with this topic since the use of carbon spikes began in 2021. They were concerned with the technical/mechanical conditions of the spikes’ construction (material, elasticity, etc.). Theoretically, the carbon sole offers a rigid base that enables runners to sustain remarkable efficiency and save vital energy during each stride [17]. Although runners’ subjective perceptions support this phenomenon, few studies [20,21] support this with objective evidence. According to some data, carbon-plated spikes likely alter the sprinting movement’s structure by strengthening the ankle joint’s stiffness [22,23,24], which produces more elastic energy [25,26,27] and thus enhances the muscles’ stretch–shortening cycle [17,18]. It has been documented that in sprints, spikes transfer more force to the ground [21,28]. This is because, in the support phase, the stiff spike sole did not change energy generation (impacted by energy restoration), and it only changed the energy lost during impact, which increases plantarflexion at the metatarsal–phalangeal (MTP) joint in the direction of toe-off [29,30,31,32]. In contrast, Mason et al. [18] stated that there is no scientific proof of how midsole thickness and material variations could affect sprinting ability when paired with a plated sole’s greater longitudinal bending stiffness. Consequently, how the new generation of sprint spikes interacts with certain sprint variables and how much they influence sprint performance is unknown.

The OptoJump Next-Microgate sensor measurement system (Optojump, Bolzano, Italy) is used to measure the essential kinematic sprinting variables. Thanks to this new technology and kinematic measurement capabilities, it is possible to check whether and how the latest carbon-plated spikes affect a sprinter’s speed capabilities. The OptoJump System’s (v.1.14.0) modular configuration, optical sensors, and LED measurement (spatial measurement accuracy of 1.41 mm and time measurement accuracy of 1 ms) enables the precise acquisition of sprint kinematic parameters [22,33,34,35]. Therefore, the main goal of this study is to determine whether the type of spike can influence the final sprint result by comparing step by step the kinematics of 50-m sprints of elite Polish juniors wearing carbon-plated and conventional spikes. Additionally, we can assume that the ground contact time and flight time, related to the recoil and accumulation of elastic energy from the rigid sole of the carbon spike, are among the predicted spatial–temporal aspects responsible for improving sprint performance.

## 2. Materials and Methods

### 2.1. Participants

Twelve well-trained junior sprinters (ages 17–19 years) from the Polish National Team (ranging from 100 to 400 m) participated in the study, with personal bests in the 100-m sprint averaging 10.70 ± 0.19 s. The athletes had an average age of 18.7 ± 1.2 years, a body height of 180.7 ± 3.5 cm, and a body mass of 73.6 ± 4.1 kg. The study occurred during the pre-competition season at the end of January 2023 as all athletes prepared for the indoor season. The tests were conducted in a 200-m indoor facility and required participants’ four to five years of consistent sprint training experience. Each participant had medical clearance to train and reported no injuries that could affect their ability to perform maximum-velocity sprints. All individuals had completed at least six months of sprint-specific strength, speed, and plyometric training before enrollment. Informed, written consent was obtained after thoroughly explaining the protocol and procedures. Additionally, parental consent was secured for those under eighteen. The Bioethics Committee for Scientific Research (3/2021) approved the protocol at the Jerzy Kukuczka Academy of Physical Education. It was conducted under the ethical standards of the Declaration of Helsinki 2013.

### 2.2. Study Design

The running protocol consisted of four 50-m all-out sprints measured under the same conditions and at the same time of day. The experimental session was conducted on an indoor-certified synthetic track. The OptoJump Next-Microgate sensor measurement system (Optojump, Bolzano, Italy) was used to measure the essential kinematic sprinting variables. The competitors ran in groups of three sprinters to best manage the five-minute rest periods between particular repetitions. Each athlete completed a standardized warm-up and baseline performance assessment, including the two 40-m 85–90% maximum-intensity accelerations. Fifty-meter sprints were completed alternately, two in classic Nike and two in the Nike Air Zoom Maxfly carbon-plated spikes. For every sprinter, the order in which the spikes were chosen was randomized. The experiment included entry spikes commercially available on the market, i.e., purchased in a store. We also confirm that Nike or another company did not sponsor the research.

### 2.3. Equipment: Sprinting Spikes

**Figure 1:**
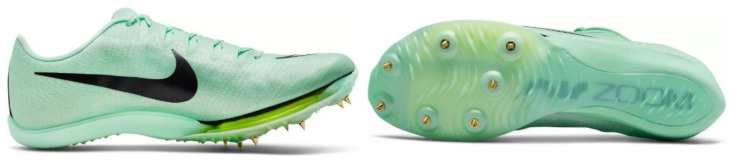
Nike ZoomX Flymax combines a PEBA plate with ZoomX foam; the plate is embedded at the heel and transitions to the spike plate (carbon fiber plates with CFPs of 13.4 and 37.1 N/mm).

For our investigation, we used commercially available Nike Air Zoom Maxfly (Figure 1) sports spikes. This spike blends ZoomX foam, which transitions to the spike plate under the forefoot, with an inserted PEBA plate at the heel. Contemporary foams, such as Nike ZoomX foam [17,36] or shoe technology [37,38], are remarkably light and sensitive, providing the best possible energy return and impact absorption. Together with a Zoom Air unit, it gives effective energy return with each stride.

### 2.4. Experimental Session

**Figure 2:**
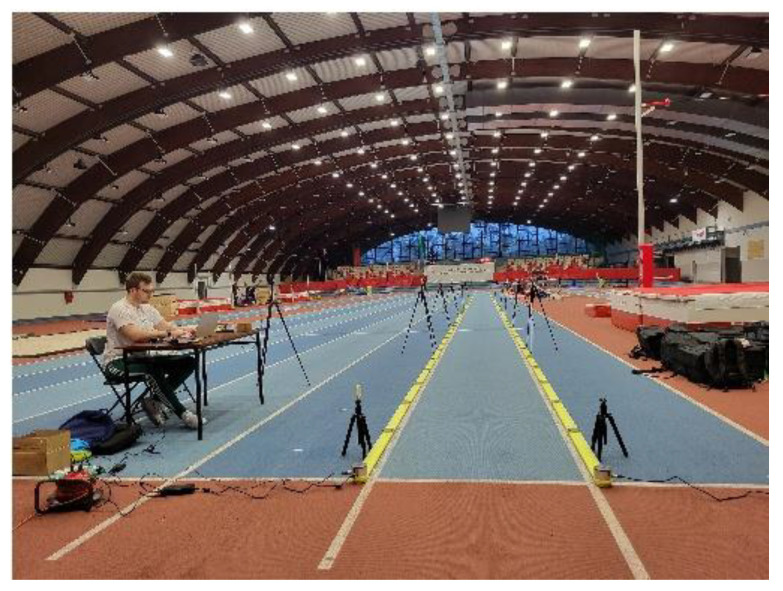
The 50-m sprint OptoJump Next System measurement station.

Six Witty cameras and the OptoJump-Microgate optical measurement system (Optojump, Bolzano, Italy) were incorporated into an indoor track for the four 50-m sprints. Following the sprint distance, the photocells were placed on the track at the start, at 10 m, at 20 m, at 30 m, and at the finish (50 m) (Figure 2). The sprinters performed their runs individually. They started from a semi-crouch position, aiming to accelerate as fast as possible to reach maximum running velocity. After two 50-m trials conducted in conventional spikes, the following two were conducted in carbon spikes, specifically the Nike Air Zoom Maxfly. A 5-min rest interval was included between each trial. The spatial–temporal properties of the twenty-two running steps, such as step length, step frequency, ground contact time, and flight duration, were measured using the OptoJump measurement system v.1.14.0. The Optojump photoelectric measurement system (Optojump-Next, Microgate, Bolzano, Italy) employs a network of connected rods with 2 bars (100 cm × 4 cm × 3 cm) that emit/receive invisible LED light beams with a grid resolution of 1 cm. Thirty-two photocells are mounted on each rod (RX bars and TX bars), spaced 4 cm apart and 0.2 cm above the surface. Each footstep interrupts the light transmission and is detected at an accuracy of 1 ms. The OptoJump system works contact-free and accurately (1000 Hz sampling rate). For processing and storing data, the gadget and computer were integrated. For analysis, all four sprint timings were used.

### 2.5. Statistical Analysis

All outcome variables were expressed using mean values, standard deviations, and 95% confidence intervals (95% CI). Cohen’s d was determined for the independent samples. Thresholds for qualitative descriptors of Cohen’s d were interpreted as ≤0.20 “small”, 0.21–0.79 “medium”, and >0.80 as “large”. The analysis was performed using a two-way repeated measures ANOVA (RM ANOVA), which considered the carbon-plated and regular spikes and the time of the four sprints. Mauchly’s test was used to assess sphericity. F-values were performed to evaluate simple main effects and interaction effects. Tukey post hoc tests were used to perform thorough comparisons. Additionally, the profile analysis process was applied. We also include ICCRs for performance testing. The coefficient for the run in classic spikes was 0.91, and for carbon spikes, it was 0.93. The three steps of the technique are as follows: (1) determining if the profiles are parallel across time points or groups; (2) determining whether the profiles have equal levels across time points or groups; and (3) determining whether the profiles are flat across time points or groups. A study was conducted on the step parameters’ internal sequence. P-values were deemed statistically significant if they were less than 0.05. All statistical analyses were performed using Statistica, Version 13 (Informer Technologies, Inc., Los Angeles, CA, USA).

## 3. Results

There was no violation of the sphericity and normalcy requirements of the residual distribution. Therefore, no revisions were made. Table 1 displays effect sizes and descriptive information for every 50 m result of spike effects.

The interaction terms and the primary effects (sprints and spikes) did not reach statistical significance. However, when the total sprint duration was considered, the sprinting impact was close to significant (F = 3.93, *p* = 0.088), and thorough comparisons using Tukey’s post hoc tests were conducted. They indicate that the total time between the first and second 50 m sprints in carbon-plated spikes had significantly improved (*p* = 0.041). The total sprint times for the first efforts (regular and carbon: *p* = 0.268) and the second sprints (*p* = 0.999) did not differ significantly. In the 50-m sprint, no discernible spike effect was present. Given that our findings showed a nonsignificant effect, a post hoc study was performed (Table 2). It seems essential to always perform pairwise group comparisons, regardless of the significance status of the omnibus test, and to report conclusions based on such group comparisons.

**Figure 3:**
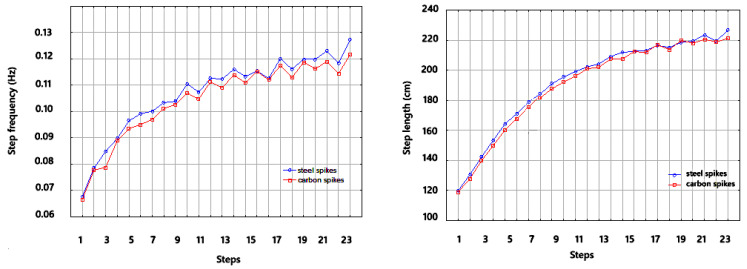
Step length profiles and flight phase duration.

The second part of the analysis was the profile analysis. Only two of the sprint step’s four primary kinematic variables—step length and flight phase time—exhibited variations between 50-m sprints conducted with different spikes. Both profiles are shown in Figure 3. Parallelism is tenable, according to the test (F = 0.68, *p* = 0.867 and F = 0.20, *p* = 0.999, respectively). The following profiles can be regarded as accidental—F = 0.28, *p* = 0.604 and F = 0.56, *p* = 0.456. This implies that discrepancies in step duration between ordinary and carbon-plated spikes carried out in twenty-four steps may be attributed to inaccuracy. Differences in the means are indicated by the test of equal means over the steps (F = 133.45, *p* < 0.001). The following step in carbon spikes was considerably longer than the preceding one in both profiles, according to post hoc tests using Tukey inequality (*p* > 0.05). Consequently, there were notable variations between steps in both profiles (F = 73.34, *p* < 0.001), and the means of the flight phase (in both profiles) did not equal the same constant. Each new step in both profiles generally took longer than the preceding one (*p* < 0.01). Between spikes, there were no appreciable variations in the same steps.

## 4. Discussion

This study’s objectives were to compare the step-by-step kinematics of elite junior Polish sprinters’ 50-m sprints conducted with carbon-plated and conventional spikes and to determine whether the type of spikes can impact a sprinter’s performance. The analysis of the 50 m sprint, performed with utmost care and thoroughness, did not show significant differences between the kinematic parameters considering runs in both the classic and carbon spikes. In conventional and carbon-plated spikes, the sprinters’ times in the second 50-m trial were faster than their initial efforts. The differences, however, were not statistically significant (*p* = 0.09), and it is possible that they represented a tendency indicating disparities in the values obtained for each of the running step’s distinct kinematic variables. In both profiles, every succeeding step was generally longer than the preceding one (*p* < 0.01). There was no discernible spike effect in the 50-m sprint since there were no appreciable variations in the same steps between spikes. Furthermore, the theoretical claims of improved sprint times from the carbon super-spikes reported in the Healey et al. [17] study did not support the results of our experiment.

One of the earliest studies conducted by Stefanyshyn and Fusco [32] found that sprinters improved their 20-m sprint time after a 20-m flying start when they attached a plate with a rigidity of 42 N/mm to their spikes. Conversely, Smith et al. [20] investigated how different sprint spike bending stiffnesses affected the maximum 40-m sprints achieved from a standing start. For instance, Nagahara et al. [21] examined 60-m sprints using spiked shoes with varying bending stiffness soles at maximum effort. These studies often focused on a limited subset of the sprint step’s kinematic properties. The application of the Opto Jump System enabled the collection of a range of kinematic characteristics throughout the 50-m sprints.

In both profiles, every succeeding step was generally longer than the preceding one (*p* < 0.01). There was no discernible spike effect in the 50-m sprint since there were no appreciable variations in the same steps between spikes. This may indicate that despite additional reinforcement of specific tendon–muscle load patterns (carbon spike sole stiffness), it does not result in beneficial mechanical changes in the sprint stride regarding the use of elastic strain energy. This phenomenon can be explained by analyzing two opposing actions: the accumulation or loss of kinetic energy [25,27,39,40,41] in the support phase of the sprint step performed both in the acceleration and top speed phases. Sprint performance depends on acceleration and maximum speed [13,42], and this is related to the magnitude of horizontal ground reaction force a sprinter can generate [22,24,43,44,45] at a specific running velocity. A sprinter’s force–velocity profile can describe this [44]. In turn, this muscle force–velocity profile can be changed by the longitudinal bending stiffness of footwear [17], followed by Takahashi et al. [46]), Beck et al. [47]), and Cigoja et al. [48]. A carbon fiber plate provides the feeling that the running step is performed dynamically (still generating a high level of force). However, the impact of increased longitudinal bending stiffness on sprinting performance remains poorly understood. Healey et al. [17], followed by Stefanyshyn and Nigg [29] in 2000 and the early publication by Nigg and Segesser [49] in 1992, state that one of the benefits of increased flexion stiffness is a reduction in the amount of energy lost at the MTP joint. This occurs during running/sprinting when the joint absorbs mechanical energy while bending in the stance phase [29,31,50]. Simultaneously, dorsiflexing for most take-off phases releases a small portion of this energy.

The lack of significant differences between spikes at ground contact may lead us to assume that the excess energy stored in the tendon’s capacity is ineffective over short distances. An extremely brief execution time of the movement structure (sprinting step), even repeated multiple times, lasting up to 10–11 s, may only utilize a limited amount of the stored elastic energy. Presumably, prolonged exercise can tap into the excess stored energy. This is supported by the absence of differences in, for instance, step frequency between regular and carbon spikes. The profiles can be considered similar (F = 0.97, *p* = 0.340). The consistency in step frequency may suggest that the same force value is applied in each step. As we know, the level of the force component influences both the frequency and the length of steps, a point confirmed by Nagahara et al. [51]. According to them, the deformation of the rigid sole of the spikes during the stance phase of the sprinting step depends on the applied force. This causes the sole of the spikes, even when very stiff, to yield towards dorsiflexion at the MTP joint during the stance phase of the sprint stride [31,52] and generates a restorative force by enhancing plantar flexion at the MTP joint [29]. Consequently, a stiffer shoe sole can effectively transfer power to the ground during the stance phase of sprinting. This suggests that spike sole stiffness may influence sprinting performance only for athletes who exert significant ground forces during the running step [53]. This was confirmed by Willwacher et al. [50], who found that footwear with greater longitudinal bending stiffness during push-off increased lever arms at the MTP and ankle joints while decreasing them at the knee joint. This is closely related to the athletes’ lower limb strength. Therefore, it can be inferred that the lower the strength of the athlete’s limbs, the less deformation of the sole occurs, resulting in a diminished capacity for producing and accumulating elastic energy. It cannot be dismissed that the athletes involved in the study lacked sufficient strength potential or were unaware of how to utilize it. Consequently, they did not achieve better results with the crimped spikes, which require a high level of force necessary to deform the sole to harness elastic energy.

In summary, the results of our investigation will certainly serve future work, which will likely investigate the effects of carbon-plated sole spikes on ground reaction forces and the accumulation/recoil of elastic energy during prolonged sprinting, e.g., 200–400 m. From the analysis of recent world-record performances of 400 m hurdles and the flat 400 m race, carbon-plated spikes can enhance performance to the highest degree during the race’s later stages when fatigue settles in the muscle–tendon complex. Neuromuscular fatigue, which reaches its peak during the last 40–50 m of the 400 m race, could modify the stretch–reflex sensitivity and decrease muscle–tendon stiffness, which, among other neuromechanical adjustments, may lead to an increase in take-off duration, thus reducing step length [54]. A diminished sensitivity of the muscle spindles and a decreased SSC affect propulsive force, which impacts sprinting velocity under extreme fatigue. The stretch reflex seems compensated by the carbon-plated spikes, which store and release mechanical energy under fatigue conditions [55].

### Limitations

There are some limitations in this study that cannot be ignored in future studies with a similar scientific problem. Twelve well-trained junior sprinters participated in the experiment, which may seem too small to achieve the optimal power of statistics. However, the sprinters belong to the Polish Junior National Team and achieve high sprint results (10.70 ± 0.19 s 100 m), guaranteeing performance quality. Another limitation was using the same spike sole stiffness value for all sprinters. Spikes with appropriate sole bending stiffness, individually adjusted to the strength of an athlete’s lower limbs, are essential for obtaining better sprint results. The above is associated with a lack of maximum force assessment (e.g., 1RM: one repetition of a maximum squat) or power (the maximal vertical ground reaction force (GRF) of one countermovement jump (CMJ) on the force plate) of the lower limbs of the surveyed sprinters. Therefore, research will be directed towards determining the effects of carbon-plated sole spikes on the utilization of the ground reaction forces through the accumulation/recoil of elastic energy during prolonged sprinting, e.g., 200–400 m. Today, implementing such a project is a huge technological challenge.

## 5. Conclusions and Practical Application

The analysis of the four 50 m sprints did not show significant differences between the kinematic parameters considering runs in both the classic Nike and carbon-plated Nike ZoomX Flymax spikes. The total time between the first and second 50 m sprints in carbon-plated spikes significantly improved (*p* = 0.041) but was not faster than in classic spikes. It may be suggested that spikes’ sole bending stiffness may not affect short-distance (up to 50–60 m) sprinting performance. Additionally, it should be assumed that young sprinters did not achieve better results in the carbon-plated sole spikes, which require a high level of force to deform the sole to obtain more elastic energy. This relationship is related to using carbon spikes with individually adjusted sole stiffness, determined by the strength of the athlete’s lower limbs. Although we did not examine the strength of the lower limbs, it seems reasonable to direct their training to increase their strength/power potential in the coming years. The last practical conclusion is that the results of our experiment can serve as guidelines for preparing research methodology assessing the effect of carbon-plated spikes on prolonged sprinting, e.g., 200–400 m, underscoring the importance of a well-designed research approach.

## Figures and Tables

**Table 1:** The descriptive statistics of the four 50-m sprints (two in conventional and two in carbon-plated spikes).

Variable	Spikes	No of Sprint	Mean	SD	95% CI		d
time (s)	regular	1	5.60	0.12	5.49	5.70	
		2	5.57	0.13	5.46	5.69	0.24
	carbon	1	5.63	0.12	5.52	5.73	0.41
		2	5.57	0.17	5.43	5.72	
number of steps (n)	regular	1	25.25	1.49	24.01	26.49	
		2	25.13	1.25	24.08	26.17	0.08
	carbon	1	25.50	1.51	24.24	26.76	0.18
		2	25.38	1.30	24.29	26.46	
length of steps (cm)	regular	1	190.59	10.25	182.02	199.15	
		2	189.86	11.03	180.64	199.09	0.22
	carbon	1	188.34	10.48	179.58	197.10	0.19
		2	191.91	10.72	182.95	200.87	
frequency of steps (Hz)	regular	1	4.57	0.29	4.32	4.81	
		2	4.60	0.30	4.35	4.86	0.07
	carbon	1	4.59	0.29	4.34	4.83	0.07
		2	4.58	0.29	4.34	4.82	
contact time (s)	regular	1	0.11	0.01	0.11	0.12	
		2	0.11	0.01	0.10	0.12	0.01
	carbon	1	0.11	0.01	0.11	0.12	0.01
		2	0.12	0.02	0.11	0.13	
flight time (s)	regular	1	0.11	0.01	0.10	0.11	
		2	0.11	0.01	0.10	0.11	0.01
	carbon	1	0.11	0.01	0.10	0.11	0.44
		2	0.12	0.03	0.09	0.15	

SD—standard deviation; CI—confidence intervals; d—Cohen effect size.

**Table 2:** The primary and combined effects of the attempt components and spikes for every outcome.

Variable	Effect	F	*p*
time	Spikes	1.04	0.341
	Run	3.93	0.088
	spikes * run	1.88	0.213
number of steps	Spikes	2.33	0.170
	Run	1.00	0.351
	spikes * run	–	–
length of steps	Spikes	0.00	0.949
	Run	1.55	0.253
	spikes * run	1.96	0.204
frequency of steps	Spikes	0.00	1.000
	Run	0.79	0.405
	spikes * run	1.42	0.272
contact time	Spikes	2.50	0.158
	Run	0.38	0.559
	spikes * run	1.01	0.349
fly time	Spikes	0.46	0.519
	Run	0.99	0.353
	spikes * run	1.18	0.313

* Describe the interaction.

## Data Availability

The data presented in this study are available on request from the corresponding author.
